# A time of transition for the *JACMP*

**DOI:** 10.1120/jacmp.v17i2.6329

**Published:** 2016-03-08

In last month's Editorial, Sam Armato and Michael Mills outlined the economic principles that drive academic journals and explained the specific model for the *JACMP* as envisioned by the Journals Business Management Committee of the American Association of Physicists in Medicine (AAPM). In this Editorial, the *JACMP* Editors explore the larger evolution in the publishing universe, the multiple transitions now facing the *JACMP*, why so many changes are happening at the same time, and what the future may look like.

Beginning in the late 1990s, the Internet created a disruptive challenge to traditional academic journal publishing. Such a challenge was overdue. Academic scientific journal publishing was and remains one of the most profitable business categories in existence, with margins of 40% and higher. Manufacturing, services, transportation, and retail operate on much smaller margins, usually well under 10%. The larger print publishers are able to command better prices for paper, printing, print advertising, and mailing costs, putting them at a significant advantage. The publishing industry underwent consolidation so that today, there are a few publishers that publish thousands of journals, with many smaller publishers either out of business or absorbed through industry consolidation. Online publishing is disruptive, in that smaller publishers can compete aggressively if they do not need to print the journal.

For many reasons, however, most academic journals were not prepared to leave the larger publishers. This reluctance is, in part, due to the quality of services provided by the publishers, the knowledge base and relationships that built the print advertising in the journal, a reluctance to absorb the inevitable loss in advertising revenue that comes with transitioning from print to electronic‐only delivery, and the lack of transparency in cost/revenue provided to the journal by the publisher. At the inception of the open access disruptive challenge, usually dated around the year 2000, it was estimated that it would take a generation for most of the print journals to disappear and to transit to online only, and for competition to chip away at the massive size of the largest print publishers. The academic publishing industry is approximately halfway through this generation. Many journals are greatly reducing or eliminating their print delivery. One business model that has emerged is to have the online repository be the “official” version of the journal. What gets printed is only a portion of the article or a portion of all articles in the online database. This preserves a significant portion of the print advertising, while reducing print and mailing costs. Another trend is the move to online advertising; at first only a slow drip, it is now more like a steady trickle. New ad delivery processes are being developed, such as the .pdf “wrapper” ads you see in *Medical Physics*. Finally, advertisers are starting to recognize that, since most research is conducted online, the only way to reach this audience is with online advertising. As such, a larger component of the advertising budget is being devoted to ads with online delivery. All of these factors make the future optimistic for online journals.

Turning our attention to the *JACMP*, it is apparent that, while many changes have already taken place, there are more changes on the horizon. The first was a change in ownership, as the Journal moved from the now defunct American College of Medical Physics (ACMP) to the AAPM in 2012. The *JACMP* now must fit into the overall publishing mission of the AAPM. The good news here is that the AAPM recognizes the value of publishing the world's leading academic clinical medical physics journal which has been associated with an Impact Factor greater than 1. The *JACMP* was recognized as a necessary complement to the *Medical Physics* journal, and its online model can be a template for some of the future changes in business model and delivery components of *Medical Physics*. In short, the *JACMP* is a good fit for the AAPM.

However, it is not practical for the *JACMP* and *Medical Physics* to be published by two separate publishers on two separate platforms. There are many reasons for this. Economy of scale dictates that the simplest and most efficient solution is a single publisher on a single platform. This has multiple advantages. Manuscripts could be easily transitioned between the two journals. Lower platform costs may be negotiated with more articles. The community of contributors will need to learn only one system, regardless of whether the article is clinical or scientific in nature. Finally, advertising products may be tailored for both journals, resulting in greater impact for the advertising dollar. These advantages are compelling, and the AAPM is currently in the process of identifying the future publisher of both its journals. The locus of this activity is the Ad Hoc Committee on Unifying Publication Platforms (AHUPP).

Once the publisher is identified and the transition to the new platform and business model is complete, what happens next? We believe the next step will be for both journals to continue the generation‐long transition to online publishing. It is very likely that print and print advertising will make up a smaller part of the future of *Medical Physics*, and perhaps the print version of *Medical Physics* could be eliminated entirely at some point. If so, what is the creative response? Is there a way for the AAPM to preserve the revenue historically received from print advertising?

All of these ideas are consistent with the continued presence and success of the *JACMP* in the publishing marketplace. To all those in the *JACMP* community, we appreciate your continued support and your many hours of volunteer efforts writing, reviewing, and editing our articles. We are hopeful that the advertising model will be profitable and robust. For those that have not yet published or reviewed for the *JACMP*, we hope that the benefits of our open access and double‐blind review process are things you will find compelling. Although the environment surrounding the Journal is changing, we intend to preserve the things that make the *JACMP* a unique presence in medical physics publishing — a rigorously peer‐reviewed journal providing open access to practical, clinically focused articles.

## COPYRIGHT

This work is licensed under a Creative Commons Attribution 4.0 International License.

Michael D. Mills, PhD

Editor‐in‐Chief, JACMP

Per H. Halvorsen, MSc

Deputy Editor‐in‐Chief, JACMP

Timothy D. Solberg, PhD

Deputy Editor‐in‐Chief, JACMP

